# Taxonomic revision of *Conobolbitina* (Bolbitiaceae, Agaricales) based on collections from Jilin Province, China

**DOI:** 10.3897/mycokeys.127.181095

**Published:** 2026-01-26

**Authors:** Han-bing Song, Hong Cheng, Yu-fei Han, Zong-long Luo, Tolgor Bau

**Affiliations:** 1 College of Agriculture and Biological Science, Dali University, Dali 671003, Yunnan, China College of Agriculture and Biological Science, Dali University Dali China; 2 Key Laboratory of Edible Fungal Resources and Utilization (North), Ministry of Agriculture and Rural Affairs, Jilin Agricultural University, Changchun 130118, Jilin, China Co-Innovation Center for Cangshan Mountain and Erhai Lake Integrated Protection and Green Development of Yunnan Province, Dali University Dali China; 3 Co-Innovation Center for Cangshan Mountain and Erhai Lake Integrated Protection and Green Development of Yunnan Province, Dali University, Dali 671003, Yunnan, China Ministry of Agriculture and Rural Affairs, Jilin Agricultural University Changchun China

**Keywords:** *
Conobolbitina
*, morphology, new taxa, phylogeny, section *Lignicola*

## Abstract

*Conobolbitina* is a morphologically diverse transitional genus within the Bolbitiaceae. However, taxonomic confusion persists, as some of its species remain classified under other genera, and its section *Verrucisporae* has been recovered as paraphyletic. This study revises the subdivision of *Conobolbitina* using integrated morphological and phylogenetic analyses of specimens from Jilin Province, China. A combined dataset of the internal transcribed spacer (ITS), nuclear large subunit ribosomal DNA (nrLSU), and translation elongation factor 1-alpha (*tef*1-α) sequences was analyzed using maximum likelihood (ML) and Bayesian inference (BI). Phylogenetic results revealed four major monophyletic clades within *Conobolbitina*, corresponding to its four sections. In sect. *Conobolbitina*, new combinations are proposed for two species, *Con. sulcata* and *Con. striipes*. In sect. *Aeruginosa*, *Con. atrocyanea* is newly combined. For sect. *Verrucisporae*, its circumscription is revised by excluding the taxa responsible for its paraphyly, and a new combination, *Con. australis*, is proposed. To accommodate the excluded taxa from sect. *Verrucisporae*, a new section, sect. *Lignicola*, is established based on morphological and phylogenetic evidence. Within this new section, three species, *Con. glutinosa*, *Con. viscosa*, and *Con. sibirica*, are newly combined, and two new species, *Con. lignicola***sp. nov**. and *Con. fuscoviolacea***sp. nov**., are described. Through these revisions, the taxonomic positions of several species are clarified, and the paraphyly of sect. *Verrucisporae* is resolved. In total, this study revises and recognizes 10 new taxonomic entities, including one new section, two new species, and seven new combinations. An identification key to the Chinese species of *Conobolbitina* is provided, along with morphological descriptions and line drawings for the two new species.

## Introduction

The genus *Conobolbitina* T. Bau & H.B. Song was established by Song and Bau (2024) to resolve the polyphyletic problem of *Pholiotina* sensu lato. Based on morphological and phylogenetic studies, the initial circumscription of *Conobolbitina* included species formerly classified under *Bolbitius* Fr., *Conocybe* Fayod, and *Pholiotina* Fayod (Song and Bau 2024). *Conobolbitina* is a transitional group, sharing characteristics with all three genera, a concept also reflected in its name, derived from “*Cono*” (*Conocybe*), “*bolbi*” (*Bolbitius*), and “*tina*” (*Pholiotina*) (Song and Bau 2024).

Song and Bau (2024) divided the genus into three sections based on the presence of a blue-green tinge on the pileus and on whether the basidiospores are verrucose or smooth: *Conobolbitina* sect. *Conobolbitina*, sect. *Aeruginosa* (Hauskn. & Krisai) T. Bau & H.B. Song, and sect. *Verrucisporae* (Singer) T. Bau & H.B. Song. Section *Conobolbitina* was originally described as *Pholiotina* sect. *Piliferae* Hauskn. & Krisai (Hausknecht 2007). However, based on differences in cheilocystidia shape, the absence of the first intron in the *tef*1-α (983–2218 bp) region in *Conocybula* T. Bau & H.B. Song, and, critically, their distinct phylogenetic positions, some species formerly placed in sect. *Piliferae* were transferred to *Conocybula* (Song and Bau 2024). Because the type species of *Conobolbitina* resides within this group, the section name is nomenclaturally established as sect. *Conobolbitina* [= sect. *Piliferae*] (Song and Bau 2024). Section *Aeruginosa* was initially described as *Pholiotina* series *Aeruginosa* Hauskn. & Krisai under sect. *Cyanopodae* Singer (Singer 1973; Hausknecht 2007). This series was later elevated to sect. *Aeruginosa* within *Conobolbitina* based on phylogenetic evidence. In addition, sect. *Cyanopodae* (Singer) T. Bau & H.B. Song is characterized by a blue coloration at the stipe base and by sub-lecythiform cheilocystidia (Song and Bau 2024). Section *Verrucisporae* was established to accommodate species with verrucose basidiospores. In the phylogeny presented by Song and Bau (2024), *Conobolbitina* sp.1 and *Conobolbitina* sp.2 both possess verrucose basidiospores, fitting the morphological definition of this section. However, their phylogenetic positions resulted in a paraphyletic grouping of sect. *Verrucisporae*, representing a current taxonomic issue within the genus (Song and Bau 2024).

Research on *Conobolbitina* in China remains limited. As of December 2025, only three confirmed species and two undetermined species have been reported from the country, with records prior to 2024 restricted to a single species (Liu 2018; Song et al. 2023; Song and Bau 2024). For instance, *Con. dasypus* (Romagn.) T. Bau & H.B. Song was initially reported from China by Liu (2018), who treated it separately as *P. utriformis* (P.D. Orton) Bon and *P. dasypus* (Romagn.) P.-A. Moreau (Bon 1991; Moreau 2005). Subsequent taxonomic revisions have demonstrated that both names are synonyms of *Con. dasypus* (Song and Bau 2024). This synonymy is supported by Arnolds (2005), who recognized *Conocybe subnuda* Kühner as a synonym of *C. utriformis* P.D. Orton, and by Hausknecht (2009), who further synonymized both *C. utriformis* and *C. subnuda* with *P. dasypus* (Romagn.) P.-A. Moreau. During his revision of Romagnesi’s *Naucoria* specimens, Moreau (2005) determined that the earliest valid description of this taxon was made by Romagnesi (1937), thereby establishing *dasypus* as the correct epithet. The transfer of this species to *Conobolbitina* was formally completed by Song and Bau (2024). The other two confirmed species in China are *Con. ochroleuca* T. Bau & H.B. Song, which has been found in both Northeast and Southwest China (e.g., collected by the authors in Dali, Yunnan, in November 2025, confirming its distribution in Southwest China; see Fig. 2), and *Con. micheliana* T. Bau & H.B. Song, which is known only from Yunnan Province (Song and Bau 2024).

**Figure 1.**
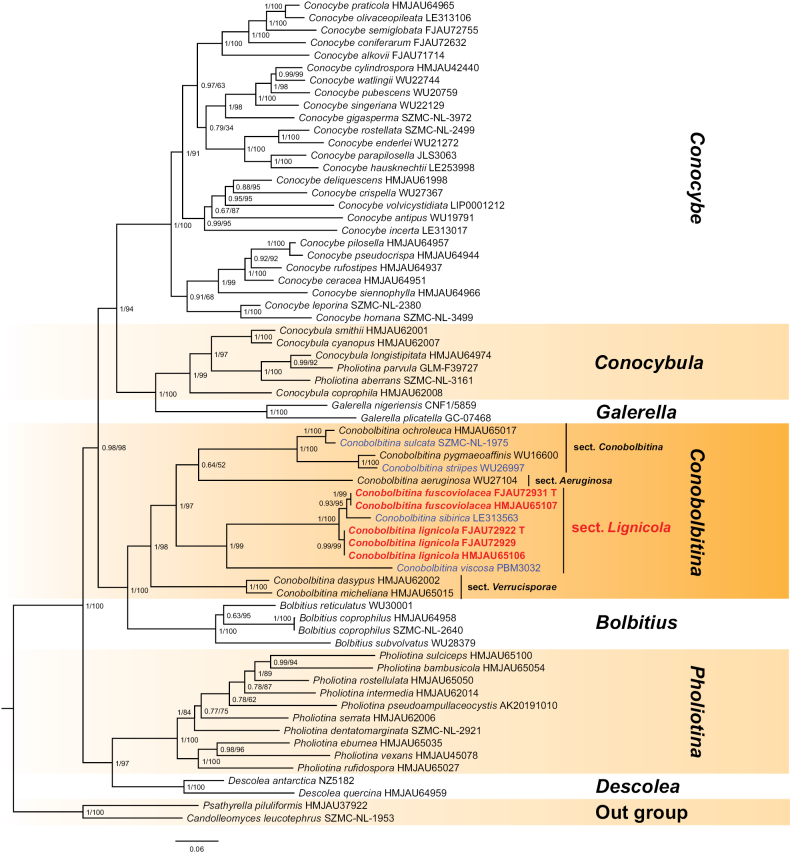
The phylogenetic relationships of *Conobolbitina* within the Bolbitiaceae family were analyzed using Bayesian inference and maximum likelihood methods based on a multi-marker dataset (ITS, nrLSU, and *tef*1-α). In the phylogenetic tree, newly proposed species are highlighted in bold red, and newly combined species are shown in blue. T = holotype.

**Figure 2.**
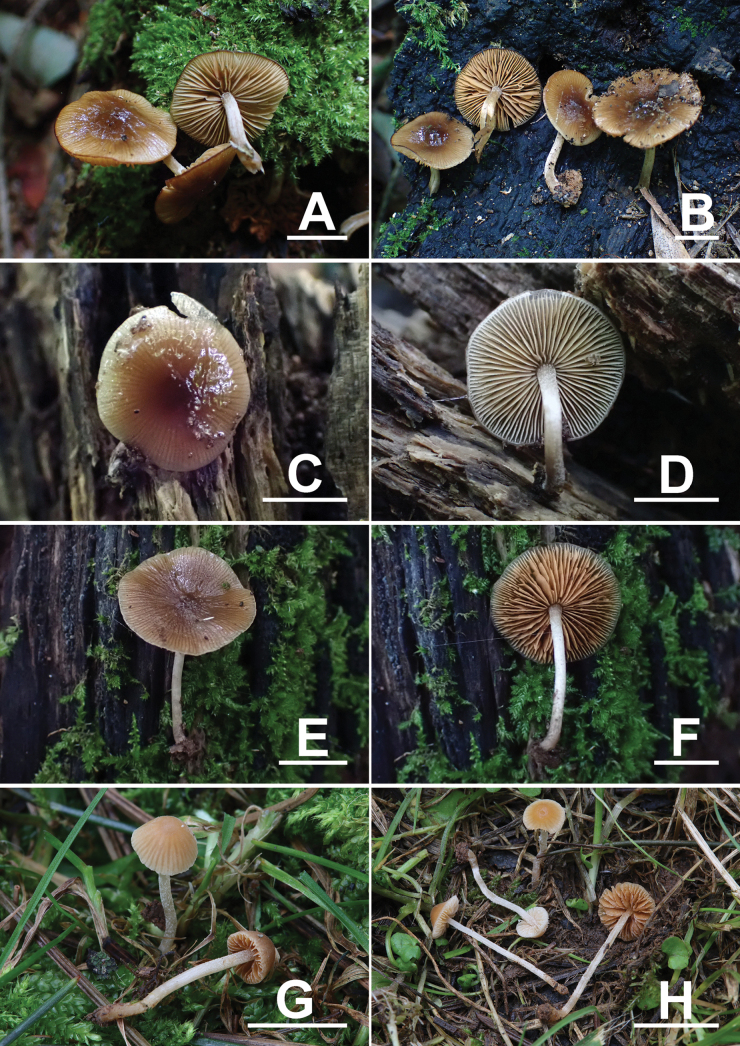
Basidiomata of *Conobolbitina* species. **A, B**. *Con. lignicola* (FJAU72922 **T**); **C, D**. *Con. lignicola* (FJAU72929); **E, F**. *Con. fuscoviolacea* (FJAU72931 **T**); **G**. *Con. ochroleuca* (FJAU72937); **H**. *Con. ochroleuca* (FJAU72951). **T** = holotype. Habitat photos of *Con. lignicola* (HMJAU65106) and *Con. fuscoviolacea* (HMJAU65107) are shown in fig. 7 of Song and Bau (2024). Scale bars: 1 cm.

The rarity of these fungi means that *Conobolbitina* sp.1 and *Conobolbitina* sp.2, which contributed to the paraphyly of sect. *Verrucisporae* were each known from only one specimen (Song and Bau 2024). Repeated annual collections were conducted at their original sites, and both taxa were successfully recollected between June and July 2025. Based on these new collections, this study aims to re-evaluate and resolve the paraphyly of sect. *Verrucisporae* propose new combinations for relevant species of *Conobolbitina* and clarify species diversity within the genus.

## Materials and methods

### Abbreviations

For Latin names: ***B .*** = *Bolbitius*; ***C.*** = *Conocybe*; ***Ca.*** = *Candolleomyces*; ***Co.*** = *Conocybula*; ***Con.*** = *Conobolbitina*; ***D.*** = *Descolea*; ***G.*** = *Galerella*; ***P.*** = *Pholiotina*; ***Po.*** = *Populus*; ***Ps.*** = *Psathyrella*.

### Samplings and morphological analyses

The specimens used in this study were collected in Jilin Province, China, between 2021 and 2025. Fresh basidiomata were photographed *in situ*, and macroscopic data were recorded, as well as geographic and ecological data, including collection site, geographic coordinates, elevation, date, habitat, and substrate. Given their typically small and delicate habitus, the basidiomata were dried rapidly using silica gel to preserve microscopic structures. Each basidioma was individually wrapped in single-ply tissue paper or corn fiber bags, placed in zip-lock bags with silica gel, and accompanied by a herbarium label. The dried specimens are deposited in the Fungarium of Jilin Agricultural University (HMJAU/FJAU).

For microscopic examination, the specimens were rehydrated in distilled water or a 5% KOH solution. When necessary, a 1% Congo red solution was applied to stain structures for improved visualization (Hausknecht 2009). Microscopic characteristics, including basidia, basidiospores, cystidia, and pileipellis elements, were observed and photographed using a Nikon Eclipse 80i microscope under oil immersion at magnifications of up to ×1000. For scanning electron microscopy (SEM) analysis, fragments of the lamellae were taken from dried specimens. These fragments were sputter-coated with gold and examined using a Hitachi TM4000 Plus II tabletop microscope at the Dali University Analysis and Testing Center. The colors of both fresh and dried basidiomata were described using the color-coding system of the German Institute for Quality Assurance and Certification (Reichs-Ausschuss für Lieferbedingungen und Gütesicherung, RAL; https://www.ral-guetezeichen.de/), abbreviated as RAL in the text.

In this study, basidiospore measurements exclude the apiculus. Measurements are expressed in the format “(a)b–c(d),” where “b–c” represents a minimum of 90% of the measured values, and “a” and “d” indicate the extremes. The dimensions of basidia, cheilocystidia, caulocystidia, and pileipellis (excluding sterigmata or excrescences) were measured when present. At least 20 structures were measured for each noted feature. The notation “(n/m/p)” indicates that measurements were taken from “n” randomly selected basidiospores obtained from “m” basidiomata belonging to “p” different collections. Twenty or 30 basidiospores were measured from each basidioma. The length-to-width ratio is referred to as Q. The mean quotient (Qm), representing the average Q, was calculated together with the standard deviation (Li et al. 2024).

### DNA extraction, PCR amplification, and sequencing

Total genomic DNA was extracted following the protocol of Song and Bau (2025). Polymerase chain reaction (PCR) amplifications were performed on a Bio-Rad T100™ Thermal Cycler (Bio-Rad Inc., Hercules, CA, USA) using the primer pairs ITS1F/ITS4 (White et al. 1990; Gardes and Bruns 1993), LR0R/LR7 (Vilgalys and Hester 1990; Moncalvo et al. 2000), and EF1-983F/EF1-2218R (Rehner and Buckley 2005) to amplify the ITS, nrLSU, and *tef*1-α gene regions, respectively. The reaction mixture composition and thermal cycling conditions followed Song and Bau (2025).

PCR products were separated by 1% agarose gel electrophoresis. Electrophoresis was conducted at 80–120 V for 30 minutes using a 2000 bp DNA ladder for size reference, and the gels were visualized under UV light. The purified PCR products were sent to Sangon Biotech (Shanghai) Co., Ltd. for sequencing. The ITS region was sequenced in one direction, whereas the nrLSU and *tef*1-α regions were sequenced in both directions and subsequently assembled.

Raw sequence chromatograms (.ab1 files) were inspected and edited using BioEdit v7.2.5 (Hall 1999). For the ITS sequences, the defined region from the 3’ end of the 18S rRNA gene (GGAAGGATCATTA) to the 5’ end of the 28S rRNA gene (TGACCTCAAA) was selected. For nrLSU and *tef*1-α sequences, low-quality terminal regions with noisy signals were trimmed. Polymorphic sites within sequences were encoded using standard IUPAC ambiguity codes (e.g., R = A/G, Y = C/T). In addition, BLAST searches were conducted against the National Center for Biotechnology Information (NCBI) database to verify the taxonomic identity of the sequences and to exclude potential contaminants. The final sequences were deposited in GenBank.

### Phylogenetic analyses

Sequence data for phylogenetic analyses were obtained from GenBank (Table 1) and supplemented with newly generated sequences from this study. The ITS, nrLSU, and *tef*1-α sequences were aligned separately using the G-INS-i algorithm implemented in the online MAFFT tool (Katoh et al. 2019; https://mafft.cbrc.jp/alignment/server/), with the maximum number of iterative refinements set to two. The resulting alignments were manually adjusted and trimmed at the ends to remove poorly aligned regions using MEGA7 (Kumar et al. 2016). A concatenated dataset was then assembled using the “Concatenate Sequence” function in PhyloSuite v2 (Zhao et al. 2025), with gaps treated as missing data (?).

**Table 1.** Information on the DNA sequences used to reconstruct phylogenetic trees. Newly generated sequences are emphasized in bold, “–” show missing sequence. T=holotype.

Taxon	Voucher specimen	GenBank accession numbers			Origin	References
		ITS	nrLSU	*tef*1-α		
*Bolbitius coprophilus*	HMJAU64958	OQ780315	OQ758216	–	China	Song and Bau 2023
*B. coprophilus*	SZMC-NL-2640	JX968253	JX968370	–	Hungary	Tóth et al. 2013
*B. reticulatus*	WU30001	JX968249	JX968366	JX968455	Hungary	Tóth et al. 2013
*B. subvolvatus*	WU28379	JX968248	JX968365	JX968454	Italy	Tóth et al. 2013
*Candolleomyces leucotephrus*	SZMC-NL-1953	FM163226	FM160683	FM897219	Hungary	Nagy et al. 2011
***Conocybe alkovii***	**FJAU71714**	**PX589399**	**PX589415**	**PX610261**	**China**	**This study**
*C. antipus*	WU19791	JX968215	JX968332	JX968432	Austria	Tóth et al. 2013
*C. ceracea*	HMJAU64951	OQ758110	OQ758218	OQ758305	China	Song and Bau 2023
***C. coniferarum***	**FJAU72632**	**PX589398**	**PX589414**	**PX610260**	**China**	**This study**
*C. crispella*	WU27367	JX968208	JX968325	JX968426	Australia	Tóth et al. 2013
*C. cylindrospora*	HMJAU42440	MG250375	OQ758203	**PX610262**	China	Liu and Bau 2018; Song and Bau 2023; **This study**
*C. deliquescens*	HMJAU61998	OP373403	OQ758204	OQ758292	China	Song and Bau 2023
*C. enderlei*	WU21272	JX968163	JX968279	–	Italy	Tóth et al. 2013
*C. gigasperma*	SZMC-NL-3972	JX968179	JX968295	JX968403	Slovakia	Tóth et al. 2013
*C. hausknechtii*	LE253789	JQ247194	–	–	Russia	Malysheva 2012
*C. hornana*	SZMC-NL-3499	JX968178	JX968294	JX968402	Slovakia	Tóth et al. 2013
*C. incerta*	LE313017	KY614062	–	–	Russia	Malysheva 2017
*C. leporina*	SZMC-NL-2380	JX968177	JX968293	JX968401	Hungary	Tóth et al. 2013
*C. olivaceopileata*	LE313106	KY614059	–	–	Russia	Malysheva 2017
*C. parapilosella*	JLS3063	MN872706	–	–	Spain	Siquier and Salom 2020
*C. pilosella*	HMJAU64957	OQ780306	OQ758206	OQ758295	China	Song and Bau 2023
*C. praticola*	HMJAU64965	OQ780303	**PX589412**	**PX610258**	China	Song and Bau 2023; **This study**
*C. pseudocrispa*	HMJAU64944	OQ780308	OQ758211	OQ758298	China	Song and Bau 2023
*C. pubescens*	WU20759	JX968170	JX968286	JX968396	Italy	Tóth et al. 2013
*C. rostellata*	SZMC-NL-2499	JX968162	JX968278	JX968390	Sweden	Tóth et al. 2013
*C. rufostipes*	HMJAU64937	OQ758120	OQ758227	OQ758317	China	Song and Bau 2023
***C. semiglobata***	**FJAU72755**	**PX589397**	**PX589413**	**PX610259**	**China**	**This study**
*C. siennophylla*	HMJAU64966	OQ780312	OQ758210	OQ758297	China	Song and Bau 2023
*C. singeriana*	WU22129	JX968166	JX968282	JX968393	Austria	Tóth et al. 2013
*C. volvicystidiata*	LIP0001212	KY346827	–	–	France	Hausknecht and Broussal 2016
*C. watlingii*	WU22744	JX968172	JX968288	JX968398	Finland	Tóth et al. 2013
*Conocybula coprophila*	HMJAU62008	OR995662	OR995712	PP000855	China	Song and Bau 2024
*Co. cyanopus*	HMJAU62007	OR995663	OR995713	PP000856	China	Song and Bau 2024
*Co. longistipitata*	HMJAU64974	OR995664	OR995714	PP000857	China	Song and Bau 2024
*Co. smithii*	HMJAU62001	OP373407	OQ758215	OQ758300	China	Song and Bau 2023; Song and Bau 2024
*Conobolbitina aeruginosa*	WU27104	JX968247	JX968364	–	Germany	Tóth et al. 2013
*Con. dasypus*	HMJAU62002	OR995661	OR995711	PP000854	China	Song and Bau 2024
***Con. fuscoviolacea***	**FJAU72931 T**	**PX612216**	**PX612213**	**PX610263**	**China**	**This study**
***Con. fuscoviolacea***	**HMJAU65107**	**OR995671**	**PP001411**	**PP000864**	**China**	Song and Bau 2024; **This study**
***Con. lignicola***	**FJAU72922 T**	**PX612217**	**PX612214**	**PX610264**	**China**	**This study**
***Con. lignicola***	**FJAU72929**	**PX612218**	**PX612215**	**PX610265**	**China**	**This study**
***Con. lignicola***	**HMJAU65106**	**OR995670**	**PP001410**	**PP000863**	**China**	Song and Bau 2024; **This study**
*Con. micheliana*	HMJAU65015	OR995677	OR994080	PP000869	China	Song and Bau 2024
*Con. ochroleuca*	HMJAU65017	OR995679	OR994082	PP000871	China	Song and Bau 2024
*Con. pygmaeoaffinis*	WU16600	JX968149	JX968382	–	Austria	Tóth et al. 2013
*Con. sibirica*	LE313563	MW682333	MW682334	–	Russia	Crous et al. 2021; **This study**
*Con. striipes*	WU26997	JX968150	JX968267	JX968383	Austria	Tóth et al. 2013; **This study**
*Con. sulcata*	SZMC-NL-1975	JX968153	JX968270	JX968386	Hungary	Tóth et al. 2013;** This study**
*Con. viscosa*	PBM3032	HQ840656	HQ840657	–	USA	Direct Submission **This study**
*Descolea antarctica*	NZ5182	AF325647	–	–	USA	Peintner et al. 2001
*D. quercina*	HMJAU64959	OQ780313	OQ758213	OQ758299	China	Song and Bau 2023
*Galerella nigeriensis*	CNF1/5859	JX968251	JX968368	JX968457	Nigeria	Tóth et al. 2013
*G. plicatella*	GC-07468	OQ845969	OR039001	–	Italy	Direct Submission
*Pholiotina aberrans*	SZMC-NL-3161	JX968256	JX968373	JX968459	Sweden	Tóth et al. 2013
*P. bambusicola*	HMJAU65054	OR995681	OR994084	PP000873	China	Song and Bau 2024
*P. dentatomarginata*	SZMC-NL-2921	JX968258	JX968374	JX968460	Hungary	Tóth et al. 2013
*P. eburnea*	HMJAU65035	OR995694	OR994097	PP000886	China	Song and Bau 2024
*P. intermedia*	HMJAU62014	OR995667	OR995717	PP000860	China	Song and Bau 2024
*P. parvula*	GLM-F39727	MK412362	–	–	Germany	Direct Submission
*P. pseudoampullaceocystis*	AK20191010	MT903471	–	–	Germany	Karich 2020
*P. rostellulata*	HMJAU65050	OR995706	OR994109	PP000898	China	Song and Bau 2024
*P. rufidispora*	HMJAU65027	OR995707	OR994110	PP000899	China	Song and Bau 2024
*P. serrata*	HMJAU62006	OP538570	OQ758217	OQ758301	China	Song and Bau 2023
*P. sulciceps*	HMJAU65100	OR995710	OR994113	PP000902	China	Song and Bau 2024
*P. vexans*	HMJAU45078	OR995669	OR995719	PP000862	China	Song and Bau 2024
*Psathyrella piluliformis*	HMJAU37922	MG734716	MW413364	MW411001	China	Yan and Bau 2018

The best-fit partitioning scheme and substitution models (edge-linked) were selected under the Akaike and Bayesian information criteria using ModelFinder v2.2.0 (Kalyaanamoorthy et al. 2017). Maximum likelihood (ML) analysis was performed using IQ-TREE 3 (Nguyen et al. 2015; Wong et al. 2025) with the selected models, and branch support was assessed using 1000 standard bootstrap replicates and the Shimodaira–Hasegawa–like approximate likelihood ratio test. Bayesian inference (BI) was conducted using MrBayes v3.2.7a (Ronquist et al. 2012) under the partitioned model, running four simultaneous Markov Chain Monte Carlo (MCMC) chains for 1,000,000 generations and sampling every 1000^th^ generation. The first 25% of trees were discarded as burn-in after confirming that the average standard deviation of split frequencies had fallen below 0.004. Phylogenetic trees were visualized and finalized using iTOL (Letunic and Bork 2019), Adobe Photoshop 2021, and Adobe Illustrator 2021. Species of *Psathyrella* and *Candolleomyces* (Song and Bau 2023) were selected as the outgroup.

## Results

### Phylogenetic analyses

The phylogenetic tree was reconstructed based on a combined dataset of ITS, nrLSU, and *tef*1-α sequences using Bayesian inference (BI). The maximum likelihood (ML) tree is not shown because it exhibited a topology consistent with the Bayesian tree. Bayesian posterior probabilities (PP) and ML bootstrap values (MLbs) are indicated at the tree nodes (Fig. 1). The multi-marker dataset (ITS + nrLSU + *tef*1-α) comprised 827 bp of ITS, 1345 bp of nrLSU, and 1102 bp of *tef*1-α. The final alignment included 66 sequences with 3274 sites, comprising 1445 distinct patterns, 1048 parsimony-informative sites, 244 singleton sites, and 1982 constant sites. For the ML analysis, the best-fit substitution models selected under the Akaike Information Criterion (AIC) were GTR+F+R4 for ITS, TIM3+F+R8 for nrLSU, and SYM+I+G4 for *tef*1-α. For the Bayesian analysis, the best-fit models selected under the Bayesian Information Criterion (BIC) were GTR+F+I+G4 for ITS and nrLSU and SYM+I+G4 for *tef*1-α.

In the phylogenetic tree (Fig. 1), new species and the new section are highlighted in bold red, whereas some newly combined species are shown in blue. With *Ps. piluliformis* (Bull.) P.D. Orton and *Ca. leucotephrus* (Berk. & Broome) D. Wächt. & A. Melzer as outgroups, all genera within Bolbitiaceae Singer formed a monophyletic group in a well-resolved tree. Among them, *Conobolbitina* and *Bolbitius* were recovered as sister groups with strong support (PP = 1, MLbs = 100).

Within *Conobolbitina*, specimens FJAU72922, FJAU72929, and HMJAU65106 formed a distinct clade, which was sister to a clade comprising *Con. sibirica* (LE313563), FJAU72931, and HMJAU65107, also with full support (PP = 1, MLbs = 100). Furthermore, FJAU72931 and HMJAU65107 formed a well-supported clade (PP = 0.93, MLbs = 95) that was sister to *Con. sibirica* (LE313563).

A BLAST search of the ITS sequence from FJAU72931 against the NCBI database revealed similarity values of 95.2% with *Con. sibirica* (LE313563), 94.1% with FJAU72922, 79.9% with *Con. viscosa* (PBM3032), and 79.37% with *Con. aeruginosa* (WU27104). For FJAU72922, the ITS sequence showed 92.8% similarity with *Con. sibirica* (LE313563), 80.2% with *Con. viscosa* (PBM3032), and 79.21% with *Con. aeruginosa* (WU27104).

Based on their distinct phylogenetic placements and morphological characteristics, two new species are proposed: *Con. lignicola* (represented by FJAU72922, FJAU72929, and HMJAU65106) and *Con. fuscoviolacea* (represented by FJAU72931 and HMJAU65107). To resolve the paraphyly of sect. *Verrucisporae*, a new section, sect. *Lignicola*, is established to accommodate the monophyletic clade comprising *Con. fuscoviolacea*, *Con. lignicola*, *Con. sibirica*, and *Con. viscosa*. This new section is sister to the clade comprising sect. *Conobolbitina* and sect. *Aeruginosa*, with strong support (PP = 1, MLbs = 97). Furthermore, seven new combinations are proposed based on their morphology or phylogenetic relationships with other species within *Conobolbitina*.

### Taxonomy

#### Conobolbitina

Taxon classificationFungiAgaricalesBolbitiaceae

T. Bau & H.B. Song, in Song & Bau, Mycosphere 15(1): 1613 (2024)

45BECCC4-0D55-5657-8C13-C9E143FC752E

##### Type species.

*Conobolbitina pygmaeoaffinis* (Fr.) T. Bau & H.B. Song.

##### Notes.

*Conobolbitina* is characterized by the absence of an annulus, a *Bolbitius*-like but non-deliquescent habit, and the presence of diverse, non-lecythiform cheilocystidia. These fungi typically grow in meadows, as well as in forested habitats on forest litter or on decaying wood. The genus has a wide global distribution (Song and Bau 2024).

#### Conobolbitina section Conobolbitina

Taxon classificationFungiAgaricalesBolbitiaceae

92D54298-8077-58B2-8BC0-3E29ED421E31

##### Synonymy.

*Pholiotina* section *Piliferae* Hauskn. & Krisai, Öst. Z. Pilzk. 16: 136 (2007).

##### Notes.

This section is characterized by the absence of a veil and any blue or blue-green coloration, smooth basidiospores, and lageniform to utriform or subcylindrical cheilocystidia with elongated necks that lack a well-defined capitulum. These fungi grow in diverse habitats, including forests, meadows, grassy roadsides, dung, and compost. The section has a broad global distribution (Song and Bau 2024).

#### Conobolbitina sulcata

Taxon classificationFungiAgaricalesBolbitiaceae

(Arnolds & Hausknecht) T. Bau & H.B. Song comb. nov.

43C39BCC-AA36-56CF-8579-79A3C6286E81

MB861426

##### Basionym.

*Pholiotina sulcata* Arnolds & Hauskn., Persoonia 18(2): 248 (2003).

##### Synonymy.

*Pholiotina sulcata* var. *oreina* Hauskn., Österr. Z. Pilzk. 16: 105 (2007).

##### Notes.

This species is characterized by smooth basidiospores and lageniform to long-necked lageniform cheilocystidia. Phylogenetic analysis confirms its placement within sect. *Conobolbitina*, based on sequence data from the specimen SZMC-NL, which is deposited in the Department of Microbiology, University of Szeged (Arnolds and Hausknecht 2003; Tóth et al. 2013).

#### Conobolbitina striipes

Taxon classificationFungiAgaricalesBolbitiaceae

(Cooke) T. Bau & H.B. Song comb. nov.

E6C0174A-6DAA-51D9-B833-93C31511A5EE

MB861429

##### Basionym.

*Agaricus striipes* Cooke [as ‘*striaepes*’], Ill. Brit. Fung. (London) 4(29/30): pl. 478 (1885).

##### Synonymy.

*Pholiotina striipes* (Cooke) M.M. Moser [as ‘*striaepes*’], in Gams, Kl. Krypt.-Fl., Edn 3 (Stuttgart) 2b/2: 229 (1967). *Conocybe striipes* (Cooke) S. Lundell [as ‘*striaepes*’], Fungi Exsiccati Suecici 41–42: 2049 (1953). *Naucoria striipes* (Cooke) Sacc. [as ‘striaepes’], Syll. fung. (Abellini) 5: 839 (1887).

##### Notes.

Both the morphological description by Hausknecht (2009) and the phylogenetic analysis by Tóth et al. (2013) support the placement of this species within sect. *Conobolbitina*.

#### Conobolbitina section Aeruginosa

Taxon classificationFungiAgaricalesBolbitiaceae

(Hauskn. & Krisai) T. Bau & H.B. Song, in Song & Bau, Mycosphere 15(1): 1616 (2024)

28240D49-FF25-50A6-963D-FC197BDD5F3B

##### Type species.

*Conobolbitina aeruginosa* (Romagn.) T. Bau & H.B. Song.

##### Basionym.

*Pholiotina* series *Aeruginosa* Hauskn. & Krisai, Öst. Z. Pilzk. 16: 135 (2007).

##### Notes.

This section is characterized by a pileus with a blue, pale greyish-blue to dark blue disc, the absence of a veil, smooth basidiospores, and lageniform to long-necked lageniform cheilocystidia (Hausknecht 2007; Song and Bau 2024).

#### Conobolbitina atrocyanea

Taxon classificationFungiAgaricalesBolbitiaceae

(Esteve-Raventós, Hausknecht & Rejos) T. Bau & H.B. Song comb. nov.

080FA383-0483-5405-A064-EAD4BF7B8094

MB861434

##### Basionym.

*Pholiotina atrocyanea* Esteve-Rav., Hauskn. & Rejos, Österr. Z. Pilzk. 16: 118 (2007).

##### Notes.

This species possesses a dark blue-green pileus, smooth basidiospores, and lageniform to sublageniform cheilocystidia, all of which align with the diagnostic characteristics of sect. *Aeruginosa* (Esteve-Raventós et al. 2007; Song and Bau 2024). This placement is consistent with its classification in the series *Aeruginosa* by Hausknecht (2009). It is worth noting that no sequence data are available to confirm this placement.

#### Conobolbitina section Verrucisporae

Taxon classificationFungiAgaricalesBolbitiaceae

(Singer) T. Bau & H.B. Song, in Song & Bau, Mycosphere 15(1): 1614 (2024)

CE90BA8F-26E0-5A17-9C8D-612C96F3BD0E

##### Type species.

*Conobolbitina verrucispora* (Singer) T. Bau & H.B. Song.

##### Basionym.

*Pholiotina* section *Verrucisporae* Singer, Beih. Sydowia 7: 79 (1973).

##### Synonymy.

*Pholiotina* subsection *Verrucisporae* (Singer) Arnolds, Persoonia 18(2): 229 (2003).

##### Description.

Based on the description in Hausknecht and Krisai-Greilhuber (2007) with revisions: Basidiomata small to medium. Pileus smooth to slightly rugulose, dry, slightly glutinous to distinctly viscid. Veil absents or weakly developed, soon vanishing. Basidiospores in light microscope almost smooth, with shallow ornamentation, slightly irregularly ridged to punctate, small to medium, with a distinct germ-pore present or absent. Cheilocystidia utriform to clavate-subcapitate. Pileocystidia present, versiform. Found in forests, on soil or litter, rarely in grassland, and almost never on wood.

##### Notes.

To resolve the paraphyly of sect. *Verrucisporae*, we have excluded those taxa characterized by a strongly viscid pileus, basidiospores with prominent, acute ornamentation, and a lignicolous habit. This taxonomic revision is supported by integrated morphological and phylogenetic evidence.

#### Conobolbitina australis

Taxon classificationFungiAgaricalesBolbitiaceae

(Singer) T. Bau & H.B. Song comb. nov.

F84C6249-09A2-5ED3-ADFF-812CE60FA5F9

MB861433

##### Basionym.

*Pholiotina australis* Singer, Beih. Nova Hedwigia 29: 214 (1969).

##### Synonymy.

*Conocybe australis* (Singer) Watling, in Watling & Gregory, Biblthca Mycol. 82: 96 (1981).

##### Notes.

This species, originally discovered in Patagonia (Argentina), was treated by Singer (1969) as a taxon closely related to *Con. dasypus* (originally described as *P. dasypus*). Horak and Hausknecht (2002) described *Con. australis* as having a vividly ochre-brown pileus, a white to pallid stipe, a fugacious veil, a subviscid pileipellis with scattered pileocystidia, and, most critically, basidiospores that are finely roughened (with insignificant ornamentation). Based on this morphological concept, we classify this species within sect. *Verrucisporae*. It is worth noting that no sequence data are available to confirm this placement.

#### Conobolbitina section Lignicola

Taxon classificationFungiAgaricalesBolbitiaceae

T. Bau & H.B. Song sect. nov.

C860C699-307E-50AA-9294-83D087508390

MB861435

##### Etymology.

“*Lignicola*” refers to the species in this section that inhabit decaying wood in forests.

##### Type species.

*Conobolbitina lignicola* T. Bau & H.B. Song.

##### Description.

Basidiomata are small to medium in size, resembling *Bolbitius* but non-deliquescent. The pileus is smooth to slightly rugulose and distinctly viscid. The veil is absent or indistinct. Basidiospores appear smooth to slightly rough under a light microscope, while scanning electron microscopy reveals prominent pointed ornamentation and irregular punctation. Basidiospores are small to medium in size, with an indistinct germ pore that may be present or absent. Cheilocystidia are diverse, non-lecythiform, and some exhibit excrescences. Caulocystidia are similar to cheilocystidia but slightly larger. Pileocystidia are present, and the species has a gelatinous layer.

##### Habitat.

Grows on decaying wood in forests.

##### Known distribution.

Asia (China, Russia), Oceania (Papua New Guinea), and North America (United States of America).

##### Notes.

This section is distinguished from sect. *Verrucisporae* by the latter’s basidiospores with shallow ornamentation and its non-lignicolous habit.

#### Conobolbitina glutinosa

Taxon classificationFungiAgaricalesBolbitiaceae

(E. Horak & Hausknecht) T. Bau & H.B. Song comb. nov.

36F8FC7E-CE6C-5887-BD12-F8582CF599CD

MB861436

##### Basionym.

*Pholiotina glutinosa* E. Horak & Hauskn., Österr. Z. Pilzk. 11: 250 (2002).

##### Notes.

Based on its lignicolous habit, viscid pileus, and basidiospores with distinct pointed ornamentation, this species is placed in sect. *Lignicola* (Horak and Hausknecht 2002).

#### Conobolbitina viscosa

Taxon classificationFungiAgaricalesBolbitiaceae

(Watling) T. Bau & H.B. Song comb. nov.

89CB540E-FFAE-5BCA-BA1C-A0BBD329CDCD

MB861453

##### Basionym.

*Bolbitius viscosus* Watling, Notes R. bot. Gdn Edinb. 34(2): 242 (1975).

##### Notes.

This species is characterized by basidiospores with distinct ornamentation and a lignicolous habit. Both its morphology and phylogenetic placement support its classification within *Conobolbitina* sect. *Lignicola* (Watling 1975).

#### Conobolbitina sibirica

Taxon classificationFungiAgaricalesBolbitiaceae

(Bulyonk., E.F. Malysheva & L.B. Kalinina) T. Bau & H.B. Song comb. nov.

91C4CA51-5462-5D03-A1B7-15836939B108

MB861454

##### Basionym.

*Bolbitius sibiricus* Bulyonk., E.F. Malysheva & L.B. Kalinina, in Crous et al., Persoonia 46: 419 (2021).

##### Notes.

The combination of morphological and phylogenetic evidence supports the placement of this species in *Conobolbitina* sect. *Lignicola* (Crous et al. 2021).

#### Conobolbitina lignicola

Taxon classificationFungiAgaricalesBolbitiaceae

T. Bau & H.B. Song sp. nov.

26CBE719-827A-5739-88E1-F008025B383B

MB861437

**Figure 3.**
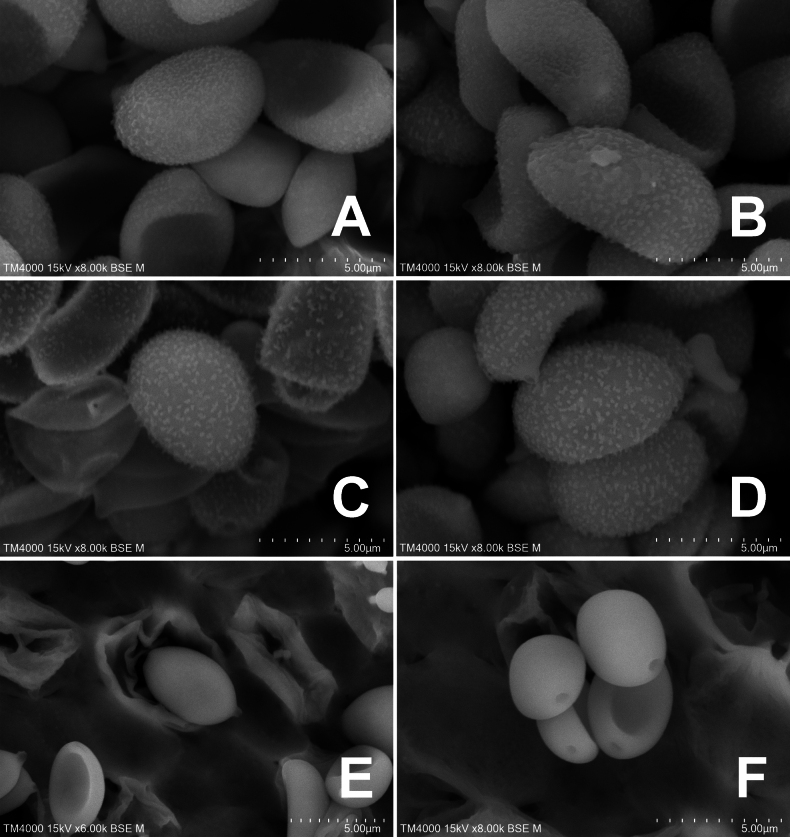
Basidiospores under electron microscopy of *Conobolbitina* species. **A, B**. *Con. lignicola* (FJAU72922 **T**); **C, D**. *Con. fuscoviolacea* (FJAU72931 **T**); **E, F**. *Con. ochroleuca* (FJAU72937). **T** = holotype. SEM images of basidiospores of *Con. lignicola* (HMJAU65106) and *Con. fuscoviolacea* (HMJAU65107) are shown in fig. 8 of Song and Bau (2024). Scale bars: 5 μm.

**Figure 4.**
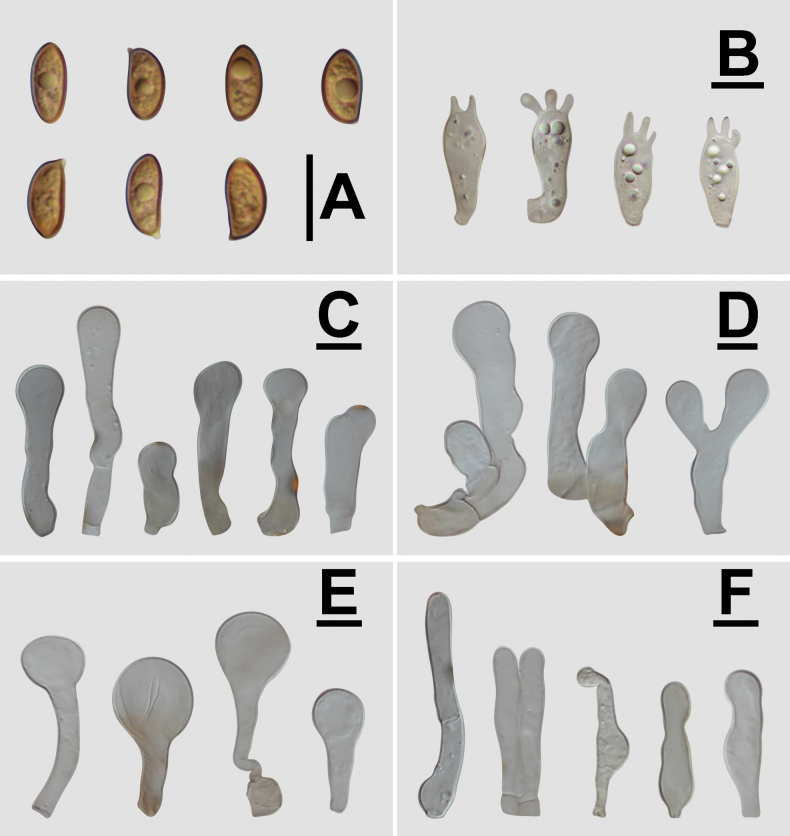
Microscopic structure images of *Conobolbitina lignicola* (FJAU72922). **A**. Basidiospores; **B**. Basidia; **C**. Cheilocystidia; **D**. Caulocystidia; **E**. Pileipellis elements; **F**. Pileocystidia. Scale bars: 10 μm.

**Figure 5.**
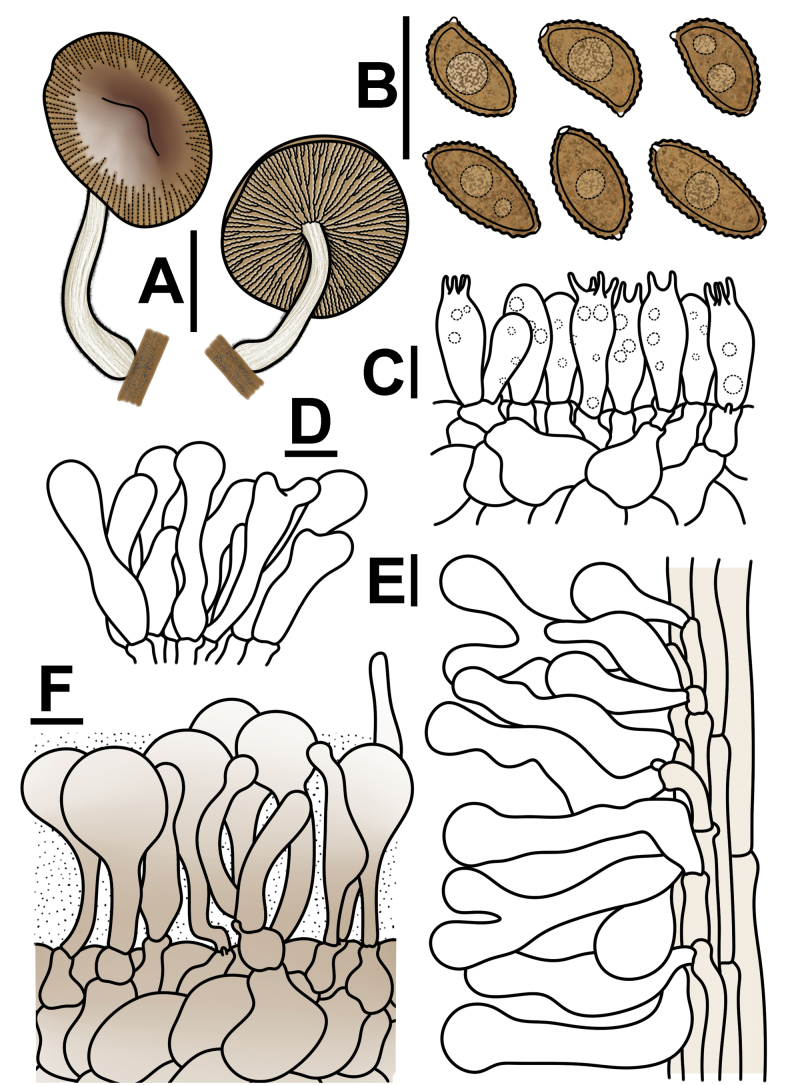
Conobolbitinalignicola (FJAU72922). **A**. Basidiomata; **B**. Basidiospores in KOH; **C**. Hymenium and subhymenium; **D**. Cheilocystidia; **E**. Stipitipellis; **F**. Pileipellis. Scale bars: 1 cm (**A**); 10 μm (**B–F**).

Figs 2A–D, 3A, 3B, 4, 5

##### Etymology.

“*lignicola*” refers to the species that inhabits decaying wood in forests.

##### Holotypus.

China • Jilin Province, Huadian City, Hongshi National Forest Park, 14 June 2025, 42°49'46"N, 127°07'48"E, alt. 454 m, T.Y. Zhang, ZTY2561407 (FJAU72922).

##### Diagnosis.

*Conobolbitina lignicola* is characterized by a subbulbous stipe base, subellipsoid to oblong basidiospores with an inconspicuous germ pore, and hyaline pileocystidia. It is further distinguished from *Con. glutinosa* by its occurrence in temperate broadleaf forests (vs. tropical rainforests of Oceania).

##### Description.

***Basidiomata*** small-sized, ***pileus*** 1–2 cm in diameter, initially broadly conical to campanulate, later straight, subumbonate, and slightly plano-concave. The pileus center is mahogany brown (RAL 8016) to chocolate brown (RAL 8017), with the margin beige (RAL 1001), sand yellow (RAL 1002) to ochre yellow (RAL 1024). The pileus is hygrophanous, smooth, viscid, with distinct striations extending up to 1/2 of the center, the margin is even to slightly undulate. ***Context*** thin, sand yellow (RAL 1002) to ochre yellow (RAL 1024), with no specific odor or taste. ***Lamellae*** adnexed to narrowly adnate, ventricose, moderately crowded, unequal in length, light ivory (RAL 1015), sand yellow (RAL 1002) to ochre brown (RAL 8001), with serrulate edges. ***Stipe*** 1–3 cm long, 2–3 mm thick, cylindrical, ivory (RAL 1014) to sand yellow (RAL 1002), covered with a powdery pubescence and longitudinally striate. The base is slightly enlarged.

***Basidiospores*** (60/3/3) (7–)7.5–10.3(–11) × (4.1–)4.3–5.6(–6) μm, Q = (1.52–)1.58–2(–2.44), Q_m_ = 1.72(±0.15), nearly ellipsoid to oblong, lemon-shaped to amygdaliform in frontal view, with a slight suprahilar depression in side view, appear smooth to slightly rough under a light microscope, with pointed ornamentation visible under a scanning electron microscope, wall thick, containing oil droplets, germ pore diameter is less than 1 μm or inconspicuous. Basidiospores are ochre brown (RAL 8001) to clay brown (RAL 8003) in KOH solution. ***Basidia*** (16–)17–23(–24) × 7–10(–11) μm, clavate, 4-spored, occasionally 2-spored, sterigmata 3–5 μm long, basidia with vacuolar contents. ***Cheilocystidia*** (15–)19–41(–42) × 6–11(–12) μm, variable in shape, cylindrical and constricted at the center, broadly capitate, narrowly utriform, broadly clavate, subcylindrical, with slightly bifurcated apices, nearly femoral head-shaped, some with excrescences, and sterile margins. ***Pleurocystidia*** absent. ***Caulocystidia*** (15–)16–61(–65) × 6–14 μm, variable in shape, similar to cheilocystidia but slightly larger, spheropedunculate, utriform, subcapitate, subclavate, subcylindrical, femoral head-shaped, with swollen and bifurcated apices, some with excrescences. ***Pileipellis*** epithelioid hymeniderm, composed of (21–)23–42(–43) × (11–)12–24(–26) μm sphaeropedunculate or broadly clavate elements, with fawn brown (RAL 8007) pigment observed in the base when viewed in KOH solution. ***Pileocystidia*** (23–)24–54(–60) × 4–10(–12) μm, variable in shape, including capitate, narrowly utriform, clavate, subcylindrical, and capilliform forms. A gelatinous layer is present. All structures have clamp connections. A weakly positive reaction with ammonia solution is observed, resulting in the formation of rhomboid crystals.

##### Habitat.

It occurs singly or scattered on decaying wood in broad-leaved forests during summer.

##### Known distribution.

Jilin Province, China.

##### Additional specimens measured.

China • Jilin Province, Jilin City, Jiaohe City, Qianjin Forest Farm, 24 June 2021, 43°57'10"N, 127°42'7"E, alt. 650 m, Q. Q. Ye, Y2408 (HMJAU65106). China • Jilin Province, Huadian City, Hongshi National Forest Park, 31 July 2025, 42°50'02"N, 127°07'58"E, alt. 487 m, H.B. Song, S2573110 (FJAU72929).

##### Notes.

This species is distinguished from *Con. glutinosa* by its occurrence in temperate broadleaf forests (vs. tropical rainforests of Oceania), a grey-brown to deep date-brown pileus, a terete stipe, basidiospores with a distinct germ pore, and smooth cystidia (Horak and Hausknecht 2002). It differs from *Con. viscosa* by the latter’s olive-black pileus with an involute margin, stipe base with pale yellow mycelium, and indistinct veil (Watling 1975). It is distinguished from *Con. sibirica* by the latter’s non-subumbonate, olive-black pileus and its weakly to strongly pigmented pileocystidia with intracellular greyish-brown granular pigment (Crous et al. 2021). Furthermore, it differs from all members of the sect. *Verrucisporae* by its *Bolbitius*-like habit, lignicolous ecology, and distinct spore ornamentation. Phylogenetically, it forms a distinct clade sister to a cluster containing *Con. sibirica* and *Con. fuscoviolacea*, with strong nodal support, and can be readily distinguished.

#### Conobolbitina fuscoviolacea

Taxon classificationFungiAgaricalesBolbitiaceae

T. Bau & H.B. Song sp. nov.

B16351CE-2C73-5336-81CA-BC318EA4AC28

MB861444

**Figure 6.**
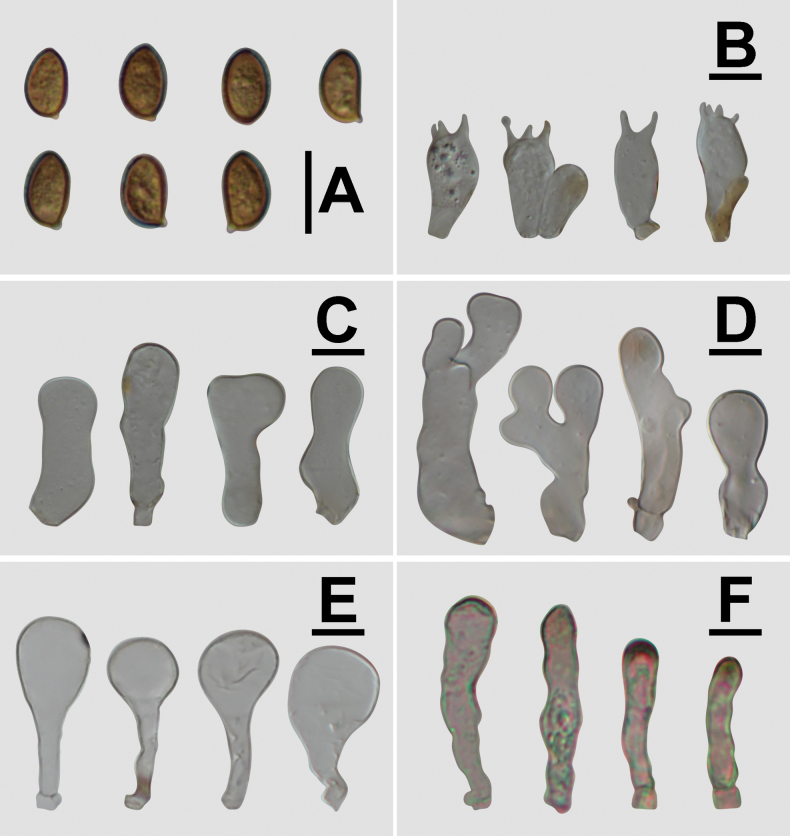
Microscopic structure images of *Conobolbitina fuscoviolacea* (FJAU72931). **A**. Basidiospores; **B**. Basidia; **C**. Cheilocystidia; **D**. Caulocystidia; **E**. Pileipellis elements; **F**. Pileocystidia. Scale bars: 10 μm.

**Figure 7.**
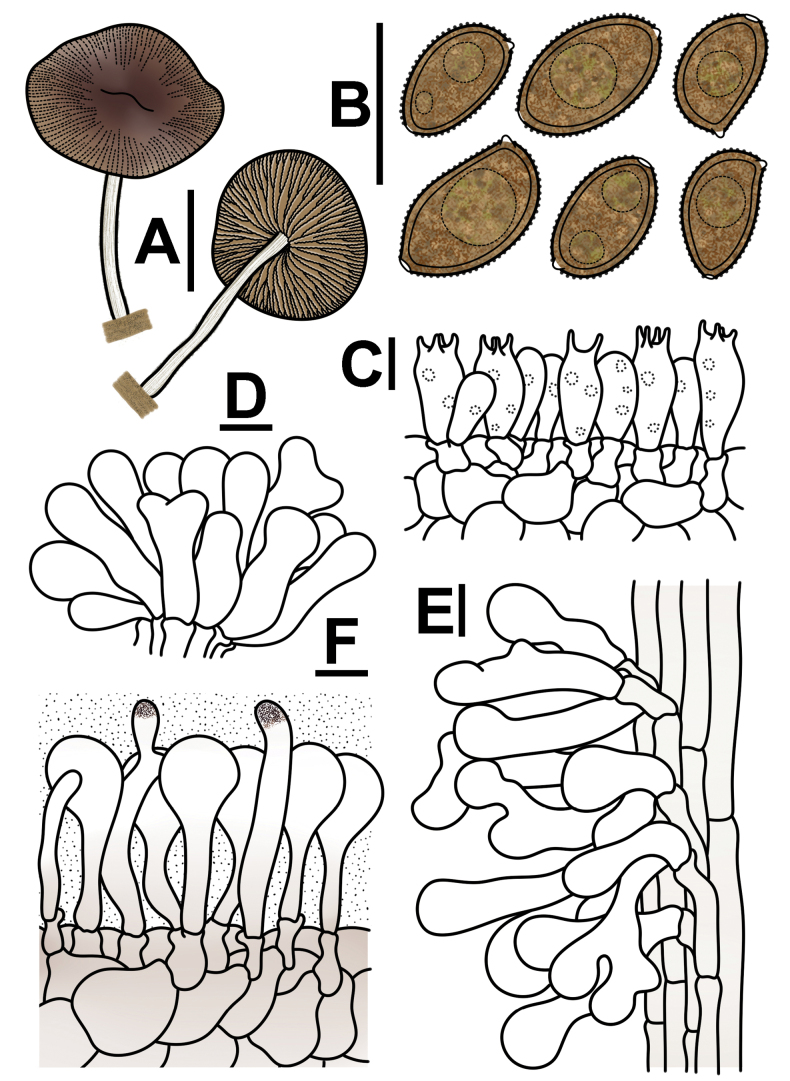
Conobolbitinafuscoviolacea (FJAU72931). **A**. Basidiomata; **B**. Basidiospores in KOH; **C**. Hymenium and subhymenium; **D**. Cheilocystidia; **E**. Stipitipellis; **F**. Pileipellis. Scale bars: 1 cm (**A**); 10 μm (**B–F**).

Figs 2E, 2F, 3C, 3D, 6, 7

##### Etymology.

“fuscoviolacea” refers to the presence of dark violet pigmentation in the pileipellis.

##### Holotypus.

China • Jilin Province, Huadian City, Hongshi National Forest Park, 31 July 2025, 42°50'01"N, 127°07'57"E, alt. 487 m, T.Y. Zhang, ZTY2573131 (FJAU72931).

##### Diagnosis.

This species is characterized by a non-bulbous stipe base, ellipsoid to oblong basidiospores, a pileipellis becoming slightly pastel violet in KOH, and pileocystidia with apical, chocolate brown to terra brown pigmentation. The combination of these features distinguishes it from other *Conobolbitina* species.

##### Description.

***Basidiomata*** small-sized. ***Pileus*** 2–3 cm in diameter, initially lentiform, later becoming straight, slightly subumbonate, plano-concave. Pileus center nut brown (RAL 8011) to sepia brown (RAL 8014), margin ivory (RAL 1014) to brown beige (RAL 1011). Pileus hygrophanous, smooth, viscid, with distinct striations extending up to 1/2 of the center; the margin even to slightly undulate. ***Context*** thin, light ivory (RAL 1015) to ivory (RAL 1014), with no specific odor or taste. ***Lamellae*** adnexed to narrowly adnate, ventricose, moderately crowded, unequal in length, beige red (RAL 3012), beige (RAL 1001) to ochre brown (RAL 8001), with serrulate edges. ***Stipe*** 2–3 cm long, 2–3 mm thick, cylindrical, light ivory (RAL 1015) to ivory (RAL 1014), surface covered with pruinose pubescence, with longitudinal fibrous striations, base is not swollen and is equal in diameter throughout.

***Basidiospores*** (60/2/2) (6.9–)7.8–10.4(–11.2) × (4.4–)4.8–6.1(–6.6) μm, Q = (1.47–)1.54–1.76(–1.78), Q_m_ = 1.65(±0.07), ellipsoid to oblong, slightly lemon-shaped in frontal view, with a slight suprahilar depression in lateral view. Appears smooth to slightly rough under a light microscope, with pointed ornamentation visible under a scanning electron microscope. Spore wall thick, containing oil droplets. Germ pore diameter less than 1.5 μm or inconspicuous. Basidiospores appear brown beige (RAL 1011) to clay brown (RAL 8003) in KOH solution. ***Basidia*** (17–)18–25 × (7–)8–11 μm, clavate, 4-spored, occasionally 2-spored, sterigmata 2–6 μm long, with vacuolar contents in basidia. ***Cheilocystidia*** (18–)22–43(–45) × 9–14(–15) μm, variable in shape, cylindrical and constricted at the center, broadly capitate, utriform, clavate, cylindrical, with bifurcated apices, nearly femoral head-shaped, some with excrescences, and sterile margins. ***Pleurocystidia*** absent. ***Caulocystidia*** (22–)23–60(–63) × 9–16 μm, variable in shape, similar to cheilocystidia but slightly larger, clavate, spheropedunculate, utriform, subcapitate, with bifurcated apices, nearly femoral head-shaped, some with excrescences. ***Pileipellis*** epithelioid hymeniderm, composed of (20–)21–42(–45) × 13–20(–21) μm sphaeropedunculate or broadly clavate elements, exhibiting red brown (RAL 8012) to pastel violet (RAL 4009) pigments. ***Pileocystidia*** (22–)24–50(–52) × 5–8 μm, clavate, subcylindrical, capilliform, some with apices pigmented chocolate brown (RAL 8017) to terra brown (RAL 8028). A gelatinous layer is present. All structures have clamp connections. A weakly positive reaction with ammonia solution is observed, resulting in the formation of rhomboid crystals.

##### Habitat.

It grows solitarily on decaying fir wood in summer.

##### Known distribution.

Jilin Province, China.

##### Additional specimens measured.

China • Jilin Province, Jilin City, Huadian City, 27 August 2023, 42°49'30"N, 127°08'17"E, alt. 483 m, H. Cheng, C2382702 (HMJAU65107).

##### Notes.

This species is distinguished from *Con. lignicola* and *Con. glutinosa* by the pastel violet reaction of its pileipellis in KOH and the chocolate brown to terra brown apical pigmentation on some pileocystidia (Horak and Hausknecht 2002). It differs from *Con. viscosa* and *Con. sibirica* by the latter two having distinctly olive-black pilei (Watling 1975; Crous et al. 2021). Phylogenetically, this species forms a sister group with *Con. sibirica*, but the latter originates from a log of *Populus tremula* L. and is phylogenetically distinct (Crous et al. 2021).

### Key to Chinese species of *Conobolbitina*

**Table:** 

1	Basidiospores smooth	***Conobolbitina ochroleuca***
–	Basidiospores rough	**2**
2	Obvious pointed ornamentation, growing on decayed wood	**3**
–	Shallow ornamentation, growing in the humus layer	**4**
3	Pileocystidia with unpigmented, Q_m_ is 1.72	***Con. lignicola***
–	Pileocystidia with chocolate-colored pigment, Q_m_ is 1.65	***Con. fuscoviolacea***
4	Pileus golden yellow	***Con. micheliana***
–	Pileus beige	***Con. dasypus***

## Discussion

Within the phylogenetic framework established by Tóth et al. (2013) and Song and Bau (2024), our analyses reveal that *Conobolbitina* forms a monophyletic group and is sister to *Bolbitius*. Furthermore, *Conobolbitina* comprises four monophyletic sections, whose phylogenetic relationships are highly consistent with their morphological characteristics.

Section *Conobolbitina* is characterized by smooth basidiospores, as observed in *Con. ochroleuca* (Fig. 3E, F). Within this section, we propose a new combination for *Con. sulcata*, a species previously classified in *Pholiotina*, a genus now restricted to taxa with a distinct annulus, a feature lacking in *Con. sulcata*. This species typically inhabits well-lit environments such as dry grasslands or nutrient-poor meadows (Arnolds and Hausknecht 2003). Its morphology conforms to sect. *Conobolbitina*, and phylogenetic analysis of the sequence *Con. sulcata* SZMC-NL-1975 confirms its placement within this section (Tóth et al. 2013). Similarly, we propose a new combination for *Con. striipes* within sect. *Conobolbitina*. This species possesses smooth basidiospores and lageniform to long-necked lageniform cheilocystidia, grows in grasslands, parks, or mixed forests, and is phylogenetically placed within this section (Moser 1967).

Section *Aeruginosa* and sect. *Conobolbitina* are sister groups. They are distinguished by the bluish-green hue of the pileus in species of sect. *Aeruginosa* (Song and Bau 2024). However, the phylogenetic support for the relationship between these two sections is relatively low (PP = 0.64, MLbs = 52). This low support may be due to the absence of a *tef*1-α sequence in the *Con. aeruginosa* dataset used for phylogenetic reconstruction and the fact that other species within this section either lack molecular data or represent undiscovered diversity. Within sect. *Aeruginosa*, we propose a new combination only for *Con. atrocyanea*. This species has a distinctly bluish-green pileus and occurs primarily in deciduous forests of the Mediterranean region, with other morphological characteristics fully conforming to the sectional definition (Esteve-Raventós et al. 2007). Its classification is further supported by Hausknecht (2009), who placed it in series *Aeruginosa*. Unfortunately, no molecular sequence data are currently available for *Con. atrocyanea*. This case highlights the critical role of morphological classification, particularly for historical species lacking sequence data. For such taxa, where type specimens are unavailable and recollection at the type locality is not feasible, taxonomic placement must rely on careful analysis of original morphological descriptions, geographical distribution, and ecological data.

Section *Lignicola* was established to resolve the paraphyly of sect. *Verrucisporae*. Species in this new section share several key characteristics that clearly distinguish them from other sections, including a *Bolbitius*-like but non-deliquescent habit, a distinctly viscid pileus, basidiospores with prominent, pointed ornamentation, and a lignicolous habit. Although species in sect. *Verrucisporae* also possess ornamented basidiospores, the ornamentation is less prominent than in sect. *Lignicola*, as illustrated in fig. 8 of Song and Bau (2024). Furthermore, species in sect. *Verrucisporae* are rarely lignicolous. Within the sect. *Lignicola*, the taxa previously designated as *Conobolbitina* sp.1 and *Conobolbitina* sp.2 in Song and Bau (2024), are here formally described as the new species *Con. lignicola* and *Con. fuscoviolacea*, respectively. *Conobolbitina lignicola* occurs on decaying wood in broad-leaved forests, whereas *Con. fuscoviolacea* is found on decaying fir wood. Several new combinations are also proposed for this section. For example, *Con. glutinosa* was transferred based on its morphological and habitat characteristics (Horak and Hausknecht 2002), although sequence data were not obtained. Its type locality is Papua New Guinea, where it was found on decaying wood in a tropical montane rainforest dominated by Fagaceae. This species was originally described in *Pholiotina*, and SEM images of its basidiospores were provided in plate 1 of Horak and Hausknecht (2002). *Conobolbitina viscosa* was originally described as a *Bolbitius* species by Watling (1975). The type specimen was collected in Emmet County, Michigan, USA, on decaying wood of *Acer* L., with SEM images of its basidiospores shown in plate 8 of Watling (1975). *Conobolbitina sibirica* was described as *B. sibiricus* by Bulyonk., Malysheva, and Kalinina in 2021 (Crous et al. 2021). Its type locality is near Novosibirsk, Russia, on a log of *Po. tremula*; SEM images of its basidiospores are included in the entry for *B. sibiricus* in Crous et al. (2021). The morphological traits and habitats of these three species are consistent with the definition of sect. *Lignicola*. Among them, *Con. viscosa* has reliable molecular sequences, and *Con. sibirica* has sequence data from the type material; phylogenetically, both are placed within the monophyletic sect. *Lignicola* clade. Accordingly, we have proposed the corresponding new combinations.

We revised the circumscription of sect. *Verrucisporae* by removing the taxa now assigned to sect. *Lignicola*. The redefined sect. *Verrucisporae* is characterized by basidiospores with relatively subtle ornamentation (see fig. 8 in Song and Bau 2024). The type species of the section is *Con. verrucispora*. Although no molecular sequence data are available for this species, its morphology, as described by Horak and Hausknecht (2002) and Hausknecht and Krisai-Greilhuber (2007), includes a dry, tomentose, and weakly translucent-striate pileus, large projecting pileocystidia, and a non-lignicolous habit, occurring on soil or litter in forests and, rarely, in grasslands. These characteristics differ markedly from those of the excluded taxa, supporting the revised sectional concept. Within this revised section, we propose a new combination for *Con. australis*. This species exhibits subtle basidiospore ornamentation and a non-lignicolous habit, consistent with the definition of sect. *Verrucisporae* (Singer 1969). Moreover, Singer (1969) considered *Con. australis* to be closely related to *Con. dasypus*. Based on this evidence, we have transferred it to sect. *Verrucisporae*, although sequence data were not obtained.

This study was prompted by the paraphyly of sect. *Verrucisporae*, which became evident through the discovery of *Conobolbitina* spp. (Song and Bau 2024). To address this issue, we first clarified the species composition of the section and proposed new combinations for several *Conobolbitina* species previously classified under other genera. Building on this framework, we revised the circumscription of sect. *Verrucisporae* by excluding the taxa responsible for the paraphyly and established a new section, sect. *Lignicola*, to accommodate them. That this taxonomic clarification was achieved despite *Conobolbitina* being a transitional genus represented by extremely scarce and rarely collected specimens further underscores the importance of morphology in fungal taxonomy.

## Supplementary Material

XML Treatment for Conobolbitina

XML Treatment for Conobolbitina section Conobolbitina

XML Treatment for Conobolbitina sulcata

XML Treatment for Conobolbitina striipes

XML Treatment for Conobolbitina section Aeruginosa

XML Treatment for Conobolbitina atrocyanea

XML Treatment for Conobolbitina section Verrucisporae

XML Treatment for Conobolbitina australis

XML Treatment for Conobolbitina section Lignicola

XML Treatment for Conobolbitina glutinosa

XML Treatment for Conobolbitina viscosa

XML Treatment for Conobolbitina sibirica

XML Treatment for Conobolbitina lignicola

XML Treatment for Conobolbitina fuscoviolacea

## Additional information

### Use of AI

No use of AI was reported.

### Author ORCIDs

Han-bing Song https://orcid.org/0000-0002-7440-5444

Hong Cheng https://orcid.org/0009-0003-0856-1048

Yu-fei Han https://orcid.org/0009-0002-4278-3341

Zong-Long Luo https://orcid.org/0000-0001-7307-4885

Tolgor Bau https://orcid.org/0000-0003-2461-9345

### Data availability

All sequences have been deposited in GenBank (https://www.ncbi.nlm.nih.gov) and MycoBank (https://www.mycobank.org). The data presented in this study are deposited in the Zenodo repository under accession number https://doi.org/10.5281/zenodo.17766165.
